# Evaluation of yield of currently available diagnostics by sample type to optimize detection of respiratory pathogens in patients with a community-acquired pneumonia

**DOI:** 10.1111/irv.12153

**Published:** 2013-08-20

**Authors:** Elisabeth G W Huijskens, John W A Rossen, Jan A J W Kluytmans, Adri G M Zanden, Marion Koopmans

**Affiliations:** a Laboratory of Medical Microbiology and Immunology, St. Elisabeth HospitalTilburg, The Netherlands; b Department of Medical Microbiology, Albert Schweitzer HospitalDordrecht, The Netherlands; c Department of Medical Microbiology, University of Groningen, University Medical Center GroningenGroningen, The Netherlands; d Laboratory for Microbiology and Infection Control, Amphia HospitalBreda, The Netherlands; e Medical Microbiology and Infection Control, VU University Medical CenterAmsterdam, The Netherlands; f Laboratory for Medical Microbiology and Public HealthEnschede, The Netherlands; g Department of Virology, Erasmus Medical CentreRotterdam, The Netherlands; h National Institute of Public Health and the Environment, Centre for Infectious Disease ControlBilthoven, the Netherlands

**Keywords:** Community-acquired pneumonia, oropharyngeal swabs, real-time PCR, respiratory virus, sputum samples, yield

## Abstract

**Background:**

For the detection of respiratory pathogens, the sampling strategy may influence the diagnostic yield. Ideally, samples from the lower respiratory tract are collected, but they are difficult to obtain.

**Objectives:**

In this study, we compared the diagnostic yield in sputum and oropharyngeal samples (OPS) for the detection of respiratory pathogens in patients with community-acquired pneumonia (CAP), with the objective to optimize our diagnostic testing algorithm.

**Methods:**

Matched sputum samples, OPS, blood cultures, serum, and urine samples were taken from patients (>18 years) with CAP and tested for the presence of possible respiratory pathogens using bacterial cultures, PCR for 17 viruses and five bacteria and urinary antigen testing.

**Results:**

When using only conventional methods, that is, blood cultures, sputum culture, urinary antigen tests, a pathogen was detected in 49·6% of patients (*n* = 57). Adding molecular detection assays increased the yield to 80%. A pathogen was detected in 77 of the 115 patients in OPS or sputum samples by PCR. The sensitivity of the OPS was lower than that of the sputum samples (57% versus 74%). In particular, bacterial pathogens were more often detected in sputum samples. The sensitivity of OPS for the detection of most viruses was higher than in sputum samples (72% versus 66%), except for human rhinovirus and respiratory syncytial virus.

**Conclusion:**

Addition of PCR on both OPS and sputum samples significantly increased the diagnostic yield. For molecular detection of bacterial pathogens, a sputum sample is imperative, but for detection of most viral pathogens, an OPS is sufficient.

## Introduction

Community-acquired pneumonia (CAP) remains a major cause of morbidity and mortality worldwide.^1^ *Streptococcus pneumoniae, Mycoplasma pneumoniae,* influenza A virus (InfA), respiratory syncytial virus (RSV), and adenoviruses (AdV) are recognized as important causes of CAP.^2^,^3^ Despite efforts to find evidence for bacterial and viral pathogens as etiological agents in patients with CAP, etiology remains elusive in up to 50% of the patients.^1^,^3^–^5^ Reasons reported for this low yield are use of antibiotics before collecting samples, sample type tested, and the diagnostic panel used for patient evaluation.^6^–^8^ Diagnostic methods used range from culture (sputum, blood, throat swabs), antigen testing (e.g. urinary antigen testing), and molecular tests. Some studies question the value of bacterial sputum culture findings.^8^–^10^ Furthermore, serologic testing requires convalescent-phase samples, and therefore, it is not useful in the initial phase of determining the etiology. Blood cultures provide a microbiological diagnosis in 0–17%, and the addition of urinary antigen detection assays for *S. pneumoniae* and *Legionella pneumophila* has improved the yield substantially.^3^,^11^ During the past years, PCR has been developed for many viral and bacterial pathogens, resulting again in higher diagnostic yields.^12^ Knowledge of the probable etiological agent(s) may inform treatment, thereby potentially reducing the use of antibiotics and eventually that of antimicrobial resistance.^10^

In this study, we aimed to assess the added value of viral and bacterial molecular diagnostics on oropharyngeal swabs (OPS) in comparison with sputum samples for the diagnosis of CAP.

## Materials and methods

### Study design

This study was embedded within a larger prospective, observational cohort study performed between April 2008 and March 2009. All patients with CAP aged 18 years and older attending the emergency ward of two teaching hospitals in Tilburg, the Netherlands, with the suspicion of CAP were included. CAP was defined as the presence of a new or progressive infiltrate on a chest radiograph with clinical symptoms suggestive of a lower respiratory tract infection. Exclusion criteria were (i) recent hospitalization (<2 weeks) or residence in long-term care facilities, (ii) known bronchial obstruction or a history of post-obstructive pneumonia (with exception of chronic obstructive pulmonary disease), (iii) primary lung cancer or another malignancy metastatic to the lungs, (iv) AIDS, known or suspected *Pneumocystis jirovecii* pneumonia, and (v) known or suspected active tuberculosis.

A case report form was obtained from all patients to collect data on age, gender, current smoking, comorbidity, clinical symptoms, prior antimicrobial treatment at admission, and blood analysis.

### Sample collection, processing, and storage

According to protocol, at the emergency ward an OPS, a sputum sample, urine sample, and a serum sample were taken and two sets of blood samples were obtained. For this comparative evaluation, only patients for whom a complete sample set was available were included. Blood and urine specimens were processed immediately. Sputum samples were divided into two equal aliquots: one for bacterial culture and another was stored at −20°C for real-time reverse transcriptase PCR ([RT]-qPCR) testing. The OPS was used to sample the posterior oropharyngeal mucosal membrane using a commercial rigid cotton-tipped swab (MWE, Wiltshire, UK). After swabbing, the OPS specimens were placed in 1·5 ml virus transport medium (Gly medium) and stored at −20°C before performing qPCR assays.

### Diagnostics

The sputum samples and blood samples were cultured according to standard microbiological procedures. All sputum samples were examined by microscopy, and sputum samples with the presence of >25 polymorphonuclear leukocytes and <10 squamous cells per field were considered to be acceptable for culture. Significant bacterial growth of the sputum sample was defined as growth of a predominant organism on the culture plates and compatible results from Gram stain.

Urine sample were tested by urinary antigen detection tests for *S. pneumoniae* and *L. pneumophila* (BinaxNOW® pneumococcal urinary antigen test and the BinaxNOW® *Legionella* urinary antigen test, Alere, Portland, ME, USA). All oropharyngeal and sputum samples were tested by (RT)-qPCR for the presence of respiratory viruses and bacteria including AdV, human bocavirus (HBoV), KI polyomaviruses and WU polyomaviruses (KIPyV and WUPyV), human metapneumovirus (hMPV), human rhinovirus (HRV), human coronaviruses (HCoV) (OC43, NL63, HKU1, and 229E), parainfluenza viruses (PIV)1−4, influenza viruses A and B (InfA, InfB), RSV, *L. pneumophila, M. pneumoniae*, *Chlamydophila psittaci, Chlamydophila pneumoniae,* and *Coxiella burnetii*. Serum samples were tested for the presence of *C. burnetii*. (RT)-qPCR procedures were performed as described.^13^–^19^ (RT)-qPCR results were expressed in cycle threshold-values.

### Statistical analysis

A consensus standard was used to assess the sensitivity of the OPS or sputum sample: A positive result in either the OPS or sputum sample was considered as the gold standard for the presence of a pathogen and was used to calculate the sensitivity of the OPS or sputum sample for the detection of the respiratory pathogens. McNemar's test was used to assess the significance of the difference between two correlated proportions. Analyses were conducted using pasw Statistics 18 (IBM Company, Chicago, VS, USA).

## Results

### Characteristics

Of the 408 patients with CAP that were evaluated during the study period, a subset of 115 (28·2%) met the inclusion criteria for completeness of sampling and was included in the study. Patients ranged in age from 20 to 90 years (mean 66 years), 62% of the patients were male. Thirty-two (27·8%) patients had had antibiotic treatment prior to admission.

### Microbiological yield

Using conventional methods, that is, blood cultures, sputum culture, urinary antigen tests, 57 patients (49·6%) tested positive for at least one pathogen. Adding the full molecular diagnostic package increased the diagnostic yield to 80%. The most frequently detected bacterial pathogens were *S. pneumoniae* (*n* = 27) and *C. burnetii* (*n* = 13). In 14 patients, *S. pneumoniae* was the only detected pathogen, and in six patients, *C. burnetii* was the only detected pathogen. The most frequently isolated viral pathogens were HRV (*n* = 13) and PIV1 (*n* = 8). In the majority of patients, HRV and PIV1 were detected in combination with other pathogens. In 58 patients (50·4%), only one pathogen was detected. Mixed infections were common, with up to three possible pathogens listed (Table 1).

**Table 1:** Diagnostic yield in 92 patients with CAP

Single pathogen (*n* = 58)		2 pathogens (*n* = 25)		3 pathogens (*n* = 9)	
14	*Streptococcus pneumoniae*	5	*S. pneumoniae*+HRV	1	*S. pneumoniae*+InfA+GNB
6	*Coxiella burnetii*	1	*S. pneumoniae*+GNB	1	*S. pneumoniae*+*Haemophilus influenzae*+RSV
6	GNB	1	*S. pneumoniae*+*C. burnetii*	1	*H. influenzae*+*C. burnetii*+HCoV OC43
5	*H. influenzae*	1	*S. pneumoniae*+InfA	1	*H. influenzae*+RSV+KIPyV
3	*Staphylococcus aureus*	1	*S. pneumoniae*+PIV1	1	*Legionella pneumophila*+PIV1+GNR
3	HRV	1	*S. pneumoniae*+HCoV OC43	1	PIV1+HRV+HCoV NL63
3	InfA	1	*S. pneumoniae*+HCoV NL63	1	InfB+WU+HCoV NL63
3	RSV	1	*L. pneumophila*+InfB	1	*S. aureus*+*P. aeruginosa*+HCoV 229E
2	*Chlamydophila psittaci*	1	*L. pneumophila*+HRV	1	*C. burnetii*+HCoV 229E+HCoV OC43
2	*L. pneumophila*	1	*L. pneumophila*+*C. burnetii*		
2	HCoV OC43	1	*L. pneumophila*+*M. pneumoniae*		
2	PIV1	1	*C. burnetii*+HRV		
1	*E. coli*	1	*C. burnetii*+*S. milleri*		
1	*Moraxella catarrhalis*	1	*C. burnetii*+*E. coli*		
1	*P. luteola*	1	*C. psittaci*+*S. aureus*		
1	AdV	1	*E. coli*+HRV		
1	InfB	1	InfA+*H. influenzae*		
1	HCoV 229E	1	PIV1+*H. influenzae*		
1	hMPv	1	PIV1+HCoV HKU		
		1	PIV1+PIV3		
		1	PIV3+HRV		

AdV, adenovirus; KIPyV, KI polyomavirus; WUPyV, WU polyomavirus; hMPV, human metapneumovirus; HRV, human rhinovirus; HCoV OC43, NL63, HKU1 and 229E, human coronaviruses; PIV1–4, parainfluenza viruses 1–4; InfA, influenza A virus; InfB, influenza B virus; RSV, respiratory syncytial virus; GNB, Gram-negative bacteria; CAP, community-acquired pneumonia.

The majority of patients with mixed infections had *S. pneumoniae* identified. *S. pneumoniae* was detected in 14 blood cultures, 20 urinary antigen tests, and five sputum samples.

**Figure 1:**
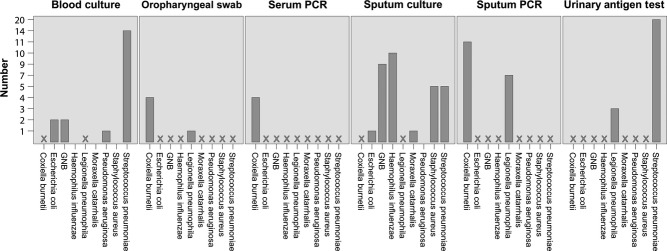
Detection of pathogens in patients with CAP by material. GNB, Gram-negative bacteria; CAP, community-acquired pneumonia; X: method not used or suitable for detection of specific pathogen.

*Haemophilus influenzae, Moraxella catarrhalis*, and *Staphylococcus aureus* were only detected in sputum cultures. *Escherichia coli* and other Gram-negative bacteria were isolated from blood cultures and sputum cultures. *Pseudomonas aeruginosa* was only isolated from a blood culture. Beside qPCR on OPS and sputum samples, *L. pneumophila* was also detected with the urinary antigen test in three patients. These patients had also a positive qPCR on OPS and/or sputum samples. *Coxiella burnetii* was detected in four serum samples; these patients had also qPCR-positive sputum samples (Figure 1).

### Sensitivity of molecular diagnostics on OPS and sputum samples

A positive qPCR in OPS and/or sputum samples was found in 77 of the 115 patients. For 33 (42·9%) of the 77 patients, the pathogens were only detected in the sputum sample, while for 20 (26·0%) of them, the pathogens were only detected in the OPS. KIPyV, WUPyV, HCoV HKU1, and HCoV 229E were only detected in the OPS, whereas *C. psittaci* was uniquely found in sputum (Table 2). The sensitivity for detecting any pathogen was 57% (95%CI: 45–68) using OPS and 74% using sputum (95%CI: 63–83). Bacterial pathogens were more often detected in sputum samples than in OPS (92%, 95%CI: 72–99 versus 25%, 95%CI: 11–47, *P* < 0·001). Except for HRV and RSV, the sensitivity for the detection of viruses using OPS was higher compared with the use of sputum samples (72%, 95%CI: 57–83 versus 66%, 95%CI: 52–78, *P* = 0·69).

**Table 2:** Detection of respiratory pathogens and the sensitivity by OPS and sputum sample

Pathogens detected in:	Both sputum and OPS	OPS only	Sputum only	Total	OPS sensitivity 95% CI	Sputum sensitivity 95% CI
Bacteria						
*Legionella pneumophila*	1	0	6	7	14 (0–58)	100 (56–100)
*Mycoplasma pneumoniae*	1	0	0	1	100 (5–100)	100 (5–100)
*Coxiella burnetii*	2	2	9	13	36 (12–68)	85 (54–97)
*Chlamydophila psittaci*	0	0	3	3	0 (0–69)	100 (31–100)
*Chlamydophila pneumoniae*	0	0	0	0	–	–
Total bacteria	4	2	18	24	25 (11–47)	92 (72– 99)
Viruses						
Adenovirus	1	0	0	1	100 (5–100)	100 (5–100)
Human bocavirus	0	0	0	0	–	–
KI polyomavirus	0	1	0	1	100 (5–100)	0 (0–95)
WU polyomavirus	0	1	0	1	100 (5–100)	0 (0–95)
Human metapneumovirus	1	0	0	1	100 (5–100)	100 (5–100)
Human rhinovirus	3	0	10	13	23 (6–54)	100 (72–100)
Human coronaviruses						
OC43	5	0	0	5	100 (46–100)	100 (46–100)
NL63	1	2	0	3	100 (31–100)	33 (2–87)
HKU1	0	1	0	1	100 (5–100)	0 (0–95)
229E	0	3	0	3	100 (31–100)	0 (0–69)
Parainfluenza viruses						
1	0	7	1	8	88 (47–99)	13 (1–53)
2	0	0	0	0	–	–
3	1	0	1	2	50 (3–97)	100 (20–100)
4	0	0	0	0	–	–
Influenza A virus	6	0	0	6	100 (52–100)	100 (52–100)
Influenza B virus	1	2	0	3	100 (31–100)	33 (2–87)
Respiratory syncytial virus	1	1	3	5	40 (7–83)	80 (30–99)
Total viruses	20	18	15	53	72 (57–83)	66 (52– 78)
Total	24	20	33	77	57 (45–68)	74 (63–83)

OPS, oropharyngeal swab, CI, confidence interval.

## Discussion

This study demonstrates that a possible etiological diagnosis can be found in a high proportion (80%) of patients with CAP, when optimal sampling and a broad diagnostic package are used.

The reality of clinical practice is that the majority of patients with CAP undergo limited diagnostic tests to demonstrate an etiological agent, other than urine antigen test and, only if available, a bacterial sputum culture. Good quality sputum samples are obtained in 40–60% of patients with CAP, but the diagnostic yield using the classical methods (culture) may be limited. In our study, a bacterial pathogen was cultured from 27% of the sputum samples, slightly higher than was found in published reports (9–14·4%).^8^,^9^,^20^

Isolation of atypical respiratory bacterial pathogens, for example *M. pneumoniae*, *C. pneumoniae* is difficult because these pathogens are hardly culturable and cell culture is time-consuming.

In our study, the use of qPCR on sputum samples increased the yield significantly for these pathogens; OPS were not suitable for the detection of them.

Respiratory viruses are poorly detected by conventional techniques.^21^ Rapid assessment of viruses is now possible with sensitive and highly specific real-time PCR assays, but the utility of swabs versus washes and nose versus oropharyngeal versus nasopharyngeal samples is subject to considerable debate. Lieberman *et al*.^22^ found that viral pathogens are better detected by nasopharyngeal washes as they offer a better yield than nasal or OPS, but this procedure is poorly tolerated and rarely used in hospitalized patients. On the other hand, de la Tabla *et al*.^23^ reported that a combined nose–throat swab was superior to nasopharyngeal aspirates for the detection of InflA (H1N1) and that the combination of both methods increases the detection rate. In our study, we used a sputum sample and OPS for the detection of viral pathogens and found that OPS was equally or more sensitive for most viruses except HRV and RSV. Falsey *et al*.^24^ found in their study that more viruses were detected in sputum samples compared with nose–throat swabs, 44% of the viruses were detected by both methods, 23% were positive by nose–throat swabs alone, and 33% were positive only with sputum samples. Similar to our study, nose–throat swabs and sputum testing yield complementary results. For bacterial pathogens, sputum samples clearly were superior to OPS. Similar to studies elsewhere, we found *S. pneumoniae* as the most common potential pathogen.^1^,^25^ In the literature, *S. pneumoniae* PCR on sputum samples as a diagnostic tool for pneumococcal CAP has had mixed results because distinguishing colonization from infection is difficult even by quantifying the load.^26^–^29^ However, patients with CAP tend to be more frequently colonized with pneumococci than asymptomatic patients and an important hypothesis is that aspiration of oropharyngeal contents the most common route is of developing pneumonia.^30^–^32^ Similarly, culturing Streptococci from sputum samples are not conclusive evidence for their etiological role. Therefore, the value of routine detection of *S. pneumoniae* remains a matter of debate. Similar to others, our study showed that molecular detection of *L. pneumophila* on sputum could replace urinary antigen testing.^33^,^34^ Practically, however, this requires a laboratory setup capable of providing such diagnostics with a rapid turnaround time, and 24/7, a situation that is currently not achievable in many settings.

In our study population, a relatively large number of *C. burnetii* in patients with CAP were found. This was due to a Q fever outbreak in our area with over 4000 notified cases in the Netherlands between 2007 and 2010.^35^ For viruses, results comparing sample types were more variable: Overall, based on our data and the convenience of the sampling procedure, OPS would be the preferred sample type, with the exception of HRV and RSV. Our findings are in agreement with published studies focusing on viral pathogens as primary causes of CAP remain an issue of considerable debate.^21^,^36^,^37^ The majority of patients with CAP positive for HRV had a second respiratory virus or a bacterial pathogen, and HCoVs were never found as a single infection. In addition, HRV are highly prevalent, and case–control studies have also found HRV to be common in asymptomatic persons as well.^21^,^36^ Our findings do suggest, however, that IF these pathogens are included in the diagnostic package and HRV testing should be integrated in a sputum panel, consisting of bacterial targets in addition to HRV and RSV. This may be more relevant for RSV for which therapeutic options are available, although the efficacy of antivirals in this patient category remains to be determined.^38^

Limitations in our study include the lack of a control group to determine the prevalence of respiratory pathogens. More than one pathogen was isolated in 34 (2·6%) of the 115 patients and in nine patients three pathogens were found. Real-time PCR significantly improves the sensitivity of detecting pathogens, and often it is not possible to determine the contribution of each pathogen as the detection of viral or bacterial nucleic acids may not always represent causation. In a study by van Gageldonk *et al*.^39^, in approximately 20% of the subjects with no respiratory complaints, respiratory viral pathogens were detected. On the other hand, Lieberman *et al*.^22^ found a much lower prevalence (7·1%) of respiratory viruses in subjects with no respiratory complaints. In this study, all subjects enrolled were symptomatic, and this would increase the likelihood that isolated pathogens were causative, unfortunately observational cohort studies such as this are not able to directly determine causation. Quantitative (RT)-qPCR data would have been useful to help address the question whether there is active infection in the lower respiratory tract instead of detecting residual DNA/RNA from a prior infection or asymptomatic carriage. Finally, we only included a subset of patients with CAP, but we have no reason to believe that the patients who were not included would be substantially different compared with the study group.

## Conclusions

Based on our findings, providing a targeted bacterial PCR package for sputum testing and a separate viral package for OPS testing would provide almost the same diagnostic yield as the full spectrum of tests used in the study. This would only be feasible if results of PCR can be available with very short turnaround time. When looking at diagnostic yield, the sputum package could include HRV and RSV testing. While this would lead to a potential diagnosis in a high proportion of CAP patients, a critical appraisal of the added value of the expanding diagnostic packages is needed, given the costs of such procedures. Studies are needed to evaluate the impact of testing algorithms on patient treatment and outcome.
